# Public views of community pharmacy services during the COVID-19 pandemic: a national survey

**DOI:** 10.1186/s40545-022-00474-4

**Published:** 2022-10-29

**Authors:** Alein W. Bou-Saba, Kassem M. Kassak, Pascale R. Salameh

**Affiliations:** 1 Doctoral School of Science and Technology, Lebanese University, Hadat, Lebanon; 2 Faculty of Health Sciences, American University of Beirut, Beirut, Lebanon; 3 Faculty of Pharmacy, Lebanese University, Hadat, Lebanon; 4 Epidémiologie Clinique et Toxicologie, Liban (INSPECT-LB), Institut National de Santé Publique, Beirut, Lebanon; 5 Department of Primary Care and Population Health, University of Nicosia Medical School, Nicosia, Cyprus; 6 School of Medicine, Lebanese American University, Byblos, Lebanon

**Keywords:** Community pharmacies, COVID-19, Good pharmacy practices, Lebanon, Public perceptions

## Abstract

**Objective:**

To assess public opinion about community pharmacy services in Lebanon during the COVID-19 pandemic.

**Method:**

A cross-sectional study using an online questionnaire was conducted between April and August of 2021. A link was shared randomly among the Lebanese population using WhatsApp and Facebook. Public perceptions were explored within 3 different indicators: general services (B) dispensing (C), and storage (D). Chi-square, Student’s test and ANOVA tests were used. *p* < 0.05 was considered statistically significant.

**Results:**

Out of 491 responses, only 9.6% scored above the 75th percentile (19.3% for the general services, 2.4% for dispensing indicator and 12.6% for storage indicator). The main concerns focused on lack of medication and reduced opening hours; however, 67.1% of respondents preferred consulting the community pharmacist instead of visiting primary health care centers, doctor’s private clinic and hospitals. Higher mean values of indicators B, C and in the overall indicator were significantly found in the presence of a pharmacist compared to the support pharmacy workforce.

**Conclusion:**

The overall public perception was inadequate. Significant difference in terms of quality of services was detected in the presence and absence of a community pharmacist during the crisis. It is recommended that the Order of Pharmacist of Lebanon (OPL) and the Ministry of Public Health (MOPH) undergo further steps mainly to enforce the laws concerning dispensing and storage indicators, improve the services in terms of extending the opening hours, ensure the availability of medicines and increase public awareness.

## Background

Health is an area where quality and safety must be the main objectives. While the quality of pharmaceutical care is defined as “the direct, responsible provision of medication-related care for the purpose of achieving definite outcomes that improve a patient’s quality of life” [1], its quality is assessed using various quality indicators [2]. Implementing quality concepts in health care services can achieve appropriate health outcomes for consumers. One of the adopted tools to assess quality is to measure customer satisfaction [3]. It is also identified that positive public perception regarding community pharmacists will enhance their role in the overall healthcare system [4]. In addition, the pharmacy profession requires a relationship of quality and trust with patients [5]. Pharmacists are in a position to inform and educate patients and are considered a frontline player in public health. The community pharmacist is available to patients daily without appointment [6]. Consequently, the pharmacist might have a positive influence and impact on the public since he/she meets and consults the highest number of persons per day compared to other health professionals [7].

In recent years, the role of the pharmacist has evolved, with pharmacist–patient communication becoming an essential element of pharmacy practice, to encourage appropriate use of drugs and achieve therapeutic success [8]. Enhancing services requires a joint contribution from pharmacists and customers; on one hand, pharmacists are encouraged to adhere to the Good Pharmacy Practices standards (GPP) that were established by the World Health Organization (WHO) and to undergo the necessary actions to promote their role, while on another hand, the patient has to accept this role and consider pharmacists’ advice regarding the offered health care services [9].

In the Middle East, previous findings showed that the public understands correctly the basic responsibilities of the community pharmacists such as medication dispensing, but did not recognize the advanced pharmaceutical services [10]. In Kuwait, people still have negative perceptions regarding community pharmacists [11]. In Lebanon, two studies [12, 13] revealed that the public perception towards the role of the community pharmacists is still poor, despite the presence of registered pharmacists.

During the COVID-19 pandemic, the community pharmacist was considered as an essential key player [14]. Some countries adapted working practices for pharmacists in order to provide continuity of services and even implemented telehealth services, set components for telehealth visits and created new procedures and technologies with audio and video capabilities to offer safe and effective services through phone calls [15]. As for Lebanon, the country has been encountering devastating economical as well as political issues for three years. Additionally, the COVID-19 pandemic has worsened the economic conditions even much more, especially after the Beirut Blast, which was considered as the third huge explosion in history. Such factors have changed people's life, so poverty is lingering in most of the Lebanese houses now. Thus, certain urgent measures should be considered seriously in order to tackle such a crisis [16, 17]. In addition, and due to the lack of many resources, it was worth assessing the delivery of services during the pandemic, since sometimes a support pharmacy workforce replaces the licensed pharmacist in his absence. These non-pharmacists’ individuals assist pharmacists in their daily practice, but their status is not regulated in Lebanon. In this country, the Order of Pharmacist (OPL) is the governing body that pharmacists have to register with before they practice the profession in the Lebanese territory and is responsible for the implementation of GPP standards. Due to the absence of issuing and regulating these standards by MOPH, the OPL has published its own GPP standards and shared them with the MOPH. However, there are no enforcing laws for their implementation so far. While the OPL laws and regulations specify that a pharmacist should be present in the pharmacy during opening hours, this rule is not enforced in Lebanon. Moreover, the law is not enforced on all prescription medications; only benzodiazepines, tramadol, products containing codeine and narcotics are stored in the safe and need a prescription; otherwise, everything else can be bought without a prescription, even antibiotics are sold without prescription regardless of a presence of a licensed pharmacist or not [18–21]. Given that pharmacy services play a vital role in public health and due to the lack of information regarding the changes related to COVID-19 and the socio-economic crisis in Lebanon, therefore, the objective of the study was to assess the current public perception toward the provision of community pharmacy services and share the responses and needs with the concerned authorities.

## Methods

### Study design and participants

This cross-sectional study included adult participants (≥ 18 years) who could read and complete an online survey in Arabic. There were no further inclusions or exclusions criteria. Data were collected over a 4-month period between April–August 2021. The online survey was developed on Lime survey platform to facilitate data collection; the survey tool and the obtained data were secured within an encrypted database via the American University of Beirut (AUB) platform. Recruitment of participants was done using a convenience sampling technique via an invitation letter sent to the public through WhatsApp. The research team used a snowball technique to fill out the survey; they sent the link on WhatsApp to family members, friends, and to large number of municipalities across Lebanon and were asked to forward the link to other friends and family members (who might not be known to the research team); the link was also shared on social media platforms such as Facebook. This maneuver was repeated until the sample size was reached. The use of WhatsApp is very common in Lebanon [22, 23] and was seen as the most appropriate method of survey distribution in order to reach a diverse sample from different regions in the country. Both Facebook and WhatsApp are social media networking services that can increase social interaction, interest, motivation, and communication, increase sense of belonging and commitment, provide rapid feedback, and enable sharing of information to a large number of people and users [24–26]. No incentive was provided to any of the participants.

### Questionnaire

The study included 2 main parts with a total of 43 items. The first part (section A) collected socio-demographic data. The second part (Section B) related to questions to assess GPP and tailored to reach the level of customer knowledge and perception [21]. The original survey tool was created by the OPL and used in a pilot study to assess Good Pharmacy Practices in community pharmacies in Lebanon but has not been validated in any other samples. The research team selected questions from this survey that explored patient safety and the quality of pharmacy practices. Indicator B for services, and included 10 items to assess availability of services, opening hours, presence of a licensed pharmacist as long as the pharmacy is open, presence of a suitable place to discuss confidential information, information available to patients, indicator C for dispensing, included 16 items to assess dispensing procedure, dispensing antibiotics without a prescription, counseling time, role of pharmacists and patient preferences during the COVID-19, and indicator D for storage included 7 items to assess storage conditions, availability of drugs and if the pharmacy is orderly stocked.

### Sample size

Sample size was calculated using Epi info program from the total Lebanese population counting 6 million and was estimated to 385 with a power of 80% and a frequency of 50% (which yield the highest sample size) and two-sided confidence level of 95%.

### Statistical analysis

Data analysis was conducted using SPSS version 25. Missing values accounted for less than 10% and were not replaced. Continuous variables were presented as mean and standard deviation, and frequency percentages for categorical variables. Internal reliability of the indicators’ questions was checked using Cronbach’s α. The Chi-square test was used to analyze the association between two categorical/nominal variables, Student’s *T* test and ANOVA were used to analyze the association between continuous and categorical variables. Absolute percentages were referred to those who answered yes, those who answered no and those who answered I don’t know. *p* < 0.05 was considered statistically significant.

## Results

The internal reliability values were as follows: 0.602 for indicator B, 0.529 for indicator C, 0.557 for indicator D and 0.687 for the overall score. The total number of responses originally was 602, which was reduced to 491 after data cleaning. Almost half of the participants (45.6%) were aged between 18 and 30 years. Females constituted 60.7% of our sample, with the highest percentage having a university degree (63.7%). Other details can be found in Table 1. Responses presented in Tables 2, 3 and 4 reflected participants who knew there was a pharmacist present.

**Table 1:** Socio-demographic characteristics of the participants

Variable	*N* (491)	Frequency (%)
Age group		
18–30	224	45.6
31–40	102	20.8
41–50	101	20.6
> 51	67	13.0
Gender		
Male	193	39.3
Female	298	60.7
Level of education		
Primary school	18	3.7
Secondary school	44	9.0
University degree	313	63.7
Post grad degree	116	23.6
Marital status		
Single	262	53.4
Married	229	46.6
Family member		
Live alone	16	3.4
2 members	37	7.5
3 members	85	17.4
4 members	151	30.8
> 4 members	201	40.9
Region		
Beirut and Mount Lebanon	200	40.7
South	211	43
Bekaa	30	6.1
North	50	10.2
Work		
Employed	163	33.2
Unemployed	328	66.8
Income per month in L.L		
< 1.000.000	151	33.2
1.000.000–2.000.000	140	30.8
2.000.000–3.000.000	71	15.6
> 3.000.000	93	20.4

**Table 2:** Bivariate analysis of indicator B and public opinion based on presence of a licensed pharmacist

Indicator B—services and facilities Answers retrieved for Yes responses (coded 1) reflecting patient satisfaction		Total	Customer feedback based on presence of licensed pharmacist (B2)			
		*N* = 491	No	Yes	I don’t Know	*p* value*
B1	Found the pharmacy closed No	177 (36.2%)	21 (11.9%)	110 (62.1%)	46 (26%)	**0.005**
B3	Presence of suitable place for confidential discussion Yes	200 (40.9%)	11 (5.5%)	131 (65.5%)	58 (29%)	**0.001**
B4	Available source of information Patient leaflet and other	247 (50.6%)	21 (8.5%)	143 (57.9%)	83 (33.6%)	0.698
B5	Availability of services Yes					
B5.1	Vaccination	146 (29.8%)	9 (6.2%)	94 (64.4%)	43 (29.4%)	**0.036**
B5.2	Blood pressure	405 (82.7%)	34 (8.4%)	232 (57.3%)	138 (34%)	0.216
B5.3	Pregnancy test	248 (50.6%)	17 (6.8%)	134 (54%)	97 (39.2%)	0.056
B5.4	HGT	294 (60%)	26 (8.8%)	170 (57.8%)	98 (33.4%)	0.551
B5.5	Glasses	145 (29.6%)	11 (7.6%)	85 (58.6%)	49 (33.8%)	0.620
	Accessibility to Yes					
B6	Seating	217 (44.6%)	9 (4.2%)	120 (55.3%)	87 (40.2%)	**0.001**
B7	Weight	401 (92.8%)	34 (8.5%)	232 (57.8%)	134 (33.4%)	0.706
B8	Height	349 (84.9%)	26 (7.4%)	207 (59.4%)	116 (33.2%)	0.068
B9	Water	109 (29.3%)	8 (7.3%)	68 (62.4%)	33 (30.3%)	0.203
B10	Toilet	88 (30.1%)	6 (6.8%)	57 (64.8%)	25 (28.4%)	0.470

Numbers in bold indicate significant *p*-values

*Pearson Chi-square test

**Table 3:** Bivariate analysis of indicator C and public opinion based on presence of licensed pharmacist

Indicator C—dispensing, preparation, administration and distribution of medicines Answers retrieved for Yes responses (coded 1) reflecting patient satisfaction		Total	Customer feedback based on presence of licensed pharmacist			*p* value*
		*N* = 491	No	Yes	I don’t know	
C1	Consult the pharmacist before buying	362 (81.9%)	32 (8.8%)	218 (60.2%)	112 (31%)	0.001
C2	Buy drugs without prescription	275 (62.2%)	24 (8.7%)	161 (58.5%)	90 (32.8%)	0.055
C3	Type of drugs bought					
C3.1	Antibiotics	116 (42.2%)	14 (12.1%)	71 (61.2%)	31 (26.7%)	0.081
C3.2	Anti-hypertensive	25 (9.1%)	3 (12%)	18 (72%)	4 (16%)	0.170
C3.3	Cardiovascular	11 (4%)	1 (9%)	6 (54.6%)	4 (36.4%)	0.961
C3.4	Anxiolytics	28 (10.2%)	4 (14.3%)	15 (53.6%)	9 (32.1%)	0.538
C3.5	Gastro-intestinal	46 (16.7%)	8 (17.4%)	28 (60.8%)	10 (21.8%)	0.033
C3.6	Neurologic	6 (2.2%)	0 (0%)	5 (83.4%)	1 (16.6%)	0.436
C3.7	Respiratory	23 (8.4%)	3 (13%)	10 (43.5%)	10 (43.5%)	0.302
C3.8	Vitamins	172 (62.5%)	19 (11%)	102 (59.3%)	51 (29.7%)	0.122
C3.9	Hormone	24 (8.7%)	5 (20.8%)	13 (54.2%)	6 (25%)	0.083
C3.10	Local	80 (29.1%)	8 (10%)	50 (62.5%)	22 (27.5%)	0.484
C3.11	Cut smoking	11 (4%)	3 (27.3%)	7 (63.6%)	1 (9.1%)	0.038
C3.12	NSAIDs	35 (12.7%)	9 (25.7%)	18 (51.5%)	8 (22.8%)	0.001
C4	Informed by the pharmacist on prescription mistake	232 (52.5%)	21 (9%)	148 (63.8%)	63 (27.2%)	0.056
C5	Explain the purpose of generic	356 (80.7%)	34 (9.5%)	207 (58.2%)	115 (32.3%)	0.003
C6	Provided with enough information to adhere to treatment	382 (86.3%)	27 (7%)	223 (58.4%)	132 (34.6%)	0.001
C7	Provided you with enough information to reduce antimicrobial resistance	330 (74.5%)	20 (6%)	193 (58.5%)	117 (35.5%)	0.001
C8	Pharmacist take into account social background, educational level, cultural beliefs, literacy, native language and mental capacity	247 (55.9%)	20 (8.1%)	158 (64%)	69 (27.9%)	0.002
C9	Consult pharmacist for unusual responses to a medicine/ treatment	313 (70.8%)	18 (5.7%)	188 (60.1%)	107 (34.2%)	0.001
C10	Pharmacist involvement Referral to doctor/stops medication	289 (92.6%)	17 (5.9%)	176 (60.9%)	96 (33.2%)	0.489
C11	Monitoring and adjustment of therapy when needed	200 (45.4%)	9 (4.5%)	120 (60%)	71 (35.5%)	0.007
C12	Given education on using web-based information resources	128 (29.1%)	7 (5.4%)	77 (60.2%)	44 (34.4%)	0.165
C13	Average time spent on counselling 1–5 mn (not included in the scoring)	311 (70.7%)	32 (10.3%)	176 (56.6%)	103 (33.1%)	0.102
C14	Medications checked when prepared by an assistant before dispensing	188 (45.3%)	4 (2.2)	127 (67.5%)	57 (30.3%)	0.001
C15	The preparatory and dispensing area in the pharmacy are Clearly clean	211 (49.6%)	11 (5.2%)	135 (63.9%)	65 (30.9%)	0.011
C16	During COVID-19 went to the pharmacist as preferences for consult	297 (67.1%)	25 (8.5%)	175 (58.9%)	97 (32.6%)	0.256

Numbers in bold indicate significant *p*-values

*Pearson Chi-square test

**Table 4:** Bivariate analysis of storage indicator and public opinion based on presence of licensed pharmacist

Indicator D: Storage Answers retrieved for Yes responses (coded 1) reflecting patient satisfaction		Total N = 491	Customer feedback based on presence of licensed pharmacist			*p* value*
			No	Yes	I don’t know	
D1	Came to the pharmacy and didn’t find medication No	86 (17.5%)	8 (9.5%)	43 (50%)	33 (38.4%)	0.624
D2	Medicines protected from direct exposure to sunlight Yes	371 (94.2%)	33 (8.9%)	219 (59%)	119 (32.1%)	0.729
D3	Presence of functional cooling system in the pharmacy Yes	351 (95.1%)	31 (8.8%)	212 (60.4%)	108 (30.8%)	0.476
D4	Presence of functional heating system in the pharmacy Yes	206 (79.2%)	18 (8.7%)	123 (59.8%)	65 (31.5%)	0.945
D5	Roof signs of leaks No	285 (100%)	25 (8.8%)	174 (61.1%)	86 (30.1%)	–
D6	All medicines are stored on shelves Yes	261 (72.9%)	24 (9.2%)	154 (59%)	83 (31.8%)	0.945
D7	Medicines, products, bottles, or containers stored on the floor Yes	40 (12.9%)	6 (15%)	21 (52.5%)	13 (32.5%)	0.275

*Pearson Chi-square test

### Services and facilities

Regarding customers perceptions towards services and facilities, 63.8% of participants found that the pharmacy was closed when they were in need for a medication and they had to move to another one. Privacy wise, 59.1% of the customers did not find a suitable place in the pharmacy for confidential discussion and 49.4% declared that there was no written information source provided about medication therapy. The most frequently received services in community pharmacies were blood pressure measurements (82.7%), hemoglucotests (60%) and pregnancy tests (50.6%). Only 29.8% confirmed the availability of vaccination services and 29.6% received optical services (eye glasses). Positive responses regarding the accessibility of seating were 40.9%, drinking water 29.3% and toilet 30.1% (Table 2).

### Dispensing medication

Approximately 82% of the participants more likely, tended to consult the pharmacist before buying the medication even if they had a medical prescription, with a significant difference (*p* < 0.001) shown in the presence of a licensed pharmacist. Almost two-thirds of the customers bought drugs without prescription (62.2%); of those, gastro-enteric, non-steroidal anti-inflammatory drugs (NSAIDS) and anti-smoking drugs were dispensed without the presence of a licensed pharmacist. Antibiotics were the most dispensed drugs without prescription (42.2%) after vitamins (62.5%) (Table 3). Of the participants, 52.5% replied that the pharmacist did inform them about the presence of mistakes in their prescription. Less than 20% of the respondents affirmed that they did not receive any information and explanation on generics and the purpose of delivering it.

A higher percentage of participants declared receiving information concerning adherence to treatment in the presence of pharmacist versus his /her absence (58.4% vs. 7%; *p* < 0.001) and pharmacists took into account the social background and educational level of customers as well as their mental capacities (*p* < 0.002). In 70.8% of the cases, customers sought the community pharmacist’s opinion for an unusual response and consultation on a medication (*p* < 0.001). Moreover, in 92.6% of the times, the pharmacists referred their customers to seek medical advice rather than intervene and stop the medication for patients’ safety matters.

For approximately half of the time (48.2%), the pharmacist performed testing to monitor and adjust therapy when needed (*p* = 0.007). Web-based information delivered to customers was still low (29.1%), and the average estimated time spent on counseling was 1–5 min (70.7%). A higher percentage of patients acknowledged finding a cleaner preparation and dispensing area in the presence of a licensed pharmacist compared to the support pharmacy workforce (*p* < 0.011). During the COVID-19 pandemic, 67.1% of patients were seeking medical advice more often in community pharmacies than in any other health care institution.

### Storage

A common problem was that pharmaceutical products were out of stock all the time (82.5%). Customers’ feedback was positive regarding the protection of drugs from direct exposure to sunlight (94%), presence of heating and cooling system ranged from 79 to 95%, absence of leaks in the roof was 100% and storing on shelves was around 73%.

### Overall indicator

**Fig. 1:**
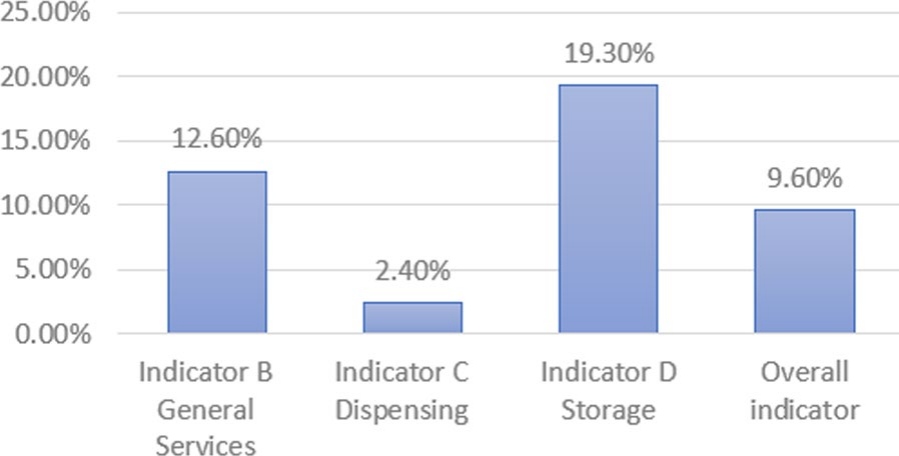
Percentage of participants who scored above 75% on Good Pharmacy Practices in Lebanon

Higher mean values of indicators B, C and in the overall indicator were significantly found in the presence of a pharmacist compared to the support pharmacy workforce (Table 5). The percentage of participants who scored above the 75% threshold was 9.6% for the overall indicator, 12.6% for indicator B, 2.4% for indicator C and 19.3% for indicator D (Fig. 1).

**Table 5:** Comparison of indicators within the presence of a licensed pharmacist and his absence and inter-item reliability testing

Indicators	Cronbach α	Presence of a pharmacy technician			Presence of a pharmacist			*p* value*
		Mean ± SD	Mean %	Min–Max	Mean ± SD	Mean %	Min–Max	
Indicator B	0.602	5.64 ± 2.25	66.7	1–11	7.06 ± 2.55	38.8	1–13	0.001
Indicator C	0.529	8.89 ± 3.50	57.8	2–17	10.25 ± 3.64	33	2–21	0.020
Indicator D	0.557	3.56 ± 1.79	44.4	0–7	4.00 ± 1.70	35.5	0–7	0.111
Overall	0.687	18.09 ± 5.55	64.4	9–32	21.31 ± 5.81	39.6	4–36	0.001

*Student’s *T* test

No significant difference was found in terms of perceptions to GPP overall score regarding age, gender, level of education, marital status, region where the pharmacy is located, work and income. A significantly higher mean score for indicator B was found in the younger age group (18–30 years) compared to the other age categories (*p* < 0.001), in those with high income compared to other categories for indicator C (*p* < 0.019), and in employed vs unemployed participants for indicator D (*p* < 0.001) (Table 6).

**Table 6:** Description of socio-demographic characteristics and association of indicators with the presence of a pharmacist reflecting customers’ perception

Socio-demographic characteristics	*N* = 491	Public perception score to GPP standards			Overall indicator/47
		Indicator B/14	Indicator C/26	Indicator D/7	
Age group		*p* < 0.001**	*p* < 0.208**	*p* < 0.205**	*p* < 0.997**
18–30	224	7.12 ± 2.46	9.32 ± 3.67	3.55 ± 1.83	19.98 ± 6.05
31–40	102	6.63 ± 2.43	9.61 ± 3.70	3.80 ± 1.80	20.04 ± 6.15
41–50	101	6.27 ± 2.51	10.00 ± 3.77	3.88 ± 1.77	20.15 ± 5.93
> 51	64	5.81 ± 2.88	10.27 ± 3.46	4.00 ± 1.69	20.08 ± 6.00
Gender		*p* < 0.991*	*p* < 0.573*	*p* < 0.278*	*p* < 0.504*
Male	193	6.67 ± 2.40	9.76 ± 3.54	3.84 ± 1.83	20.26 ± 5.84
Female	298	6.67 ± 2.66	9.57 ± 3.76	3.66 ± 1.77	19.90 ± 6.14
Level of education		*p* < 0.367**	*p* < 0.459**	*p* < 0.553**	*p* < 0.334**
8 years in school	18	5.83 ± 2.85	8.44 ± 3.76	3.22 ± 1.55	17.50 ± 6.53
Finished school	44	6.32 ± 2.62	10.07 ± 3.76	3.64 ± 1.97	20.02 ± 5.83
University degree	313	6.76 ± 2.51	9.61 ± 3.75	3.73 ± 1.85	20.10 ± 6.08
Post grad degree	116	6.68 ± 2.62	9.74 ± 3.42	3.85 ± 1.60	20.28 ± 5.83
Marital status		*p* < 0.094*	*p* < 0.201*	*p* < 0.107*	*p* < 0.583*
Single	262	6.85 ± 2.54	9.44 ± 3.68	3.61 ± 1.80	19.90 ± 6.00
Married	229	6.46 ± 2.57	9.87 ± 3.67	3.87 ± 1.79	20.20 ± 6.05
Family member		*p* < 0.092**	*p* < 0.243**	*p* < 0.717**	*p* < 0.303**
Live alone	16	5.00 ± 2.94	8.81 ± 4.32	3.63 ± 1.92	17.44 ± 7.36
2 members	37	6.70 ± 2.30	10.08 ± 3.51	3.70 ± 1.91	20.49 ± 5.90
3 members	85	6.86 ± 2.63	9.34 ± 3.81	3.69 ± 1.75	19.89 ± 6.14
4 members	151	6.58 ± 2.56	10.13 ± 3.64	3.90 ± 1.76	20.61 ± 5.94
> 4 members	201	6.80 ± 2.51	9.39 ± 3.62	3.63 ± 1.82	19.82 ± 5.94
Region		*p* < 0.135**	*p* < 0.570**	*p* < 0.817**	*p* < 0.291**
Beirut and Mount	200	6.66 ± 2.59	8.81 ± 4.32	3.79 ± 1.74	17.44 ± 7.36
Lebanon	211	6.72 ± 2.50	10.08 ± 3.51	3.73 ± 1.84	20.49 ± 5.90
South	30	7.43 ± 2.67	9.34 ± 3.81	3.60 ± 1.69	19.89 ± 6.14
Bekaa	50	6.06 ± 2.55	10.13 ± 3.64	3.54 ± 1.76	20.61 ± 5.94
North					
Work		*p* < 0.102*	*p* < 0.083*	*p* < 0.001*	*p* < 0.179*
Unemployed	163	6.94 ± 2.49	9.23 ± 3.63	3.35 ± 1.79	19.52 ± 5.68
Employed	328	6.54 ± 2.58	9.84 ± 3.68	3.92 ± 1.77	20.30 ± 6.18
Income		*p* < 0.985**	*p* < 0.019**	*p* < 0.093**	*p* < 0.07**3
< 1.000.000	151	6.58 ± 2.48	9.28 ± 3.72	3.50 ± 1.89	19.36 ± 6.16
1.000.000–2.000.000	140	6.66 ± 2.60	9.38 ± 3.72	3.71 ± 1.64	19.74 ± 6.12
2.000.000–3.000.000	71	6.62 ± 2.59	10.86 ± 3.16	4.14 ± 1.77	21.62 ± 5.27
> 3.000.000	93	6.71 ± 2.67	9.52 ± 3.83	3.83 ± 1.81	20.05 ± 6.38

*Student’s *T* test; **ANOVA

## Discussion

To our knowledge, this is the first study in Lebanon that assessed pharmacy practices from a public perception during the COVID-19 pandemic. Concerns regarding the availability of services, as indicated by the majority of participants were lack of medicines, pharmacy closure, reduced time of opening hours, unavailability of a suitable place to discuss confidential information, absence of written resources of information, inaccessibility to seating, drinking water and toilets. These issues have also been raised in May 2022 in a document published by the OPL entitled: “Towards a national pharmaceutical strategy in Lebanon” [27]. The shortages of medicines and pharmacies closure were also highly reported in the Lebanese social media [28, 29]. This is a subject of an increasing concern not only to professionals, but also to patients. The shortage of certain drugs is becoming problematic, with possible consequences and safety risks for patients. Although the risk still exists in therapeutic alternatives since physicians can replace a drug by another, however, the new drug might be less effective, have more side effects, or sometimes both [30]. Consequently, certain mechanisms are encouraged to be employed by the MOPH and the OPL. Mechanisms must ensure that emergency preparedness procedures are developed, activated and validated and that medicinal products are affordable on the Lebanese market. Such procedures must cover the whole process from importation of pharmaceutical products, to storage and distribution practices until reaching the Lebanese community pharmacies to ensure that quality and safe medications is available for all in an equitable manner. Moreover, it is worth mentioning that the shortage in medicines in pharmacies was not only due to COVID-19 pandemic as in the rest of the world, but was exacerbated by the financial crisis in Lebanon which has played an essential role as well.

One area to be also improved relates to dispensing medications without a prescription, especially antibiotics. One study in Saudi Arabia showed that reasons behind such behavior are that (1) patients presented lack of willingness to consult a physician if there is no serious infection (69.9%) and (2) they cannot afford the physician’s consultation fee (65.3%) [31]. In such case, the public has to be provided with the adequate information about the importance of adherence and completion of the full course of antibiotics, since selling antibiotics without a prescription could lead to irrational drug use and antibiotic resistance [32–34]. It could be possible that pharmacists were approached more often for the supply of antibiotics during this period. Further research should be carried out to explore whether pharmacies sale and supply of antibiotics derived from the effects of the pandemic or were a cause of concern more generally. Moreover, further investigation should tackle and weigh the influence on these selling on patient perception and satisfaction and health status. Furthermore, it is highly recommended that the MOPH imposes the laws of selling antibiotics, and other prescription medications, without a valid prescription. Concerning generic substitution, the results suggested to improve the drug substitution policy and its implementation thus, similar to recommendations of a study carried in Lebanon [35].

As for counselling, most of the respondents (70.8%) consult the pharmacist for unusual responses to a medicine or a treatment and stated that during the consultation, the pharmacist takes into account the social background, educational level, cultural beliefs, literacy, native language and mental capacity. These findings might reveal the public trust in the community pharmacist. Little literature is available on how trust can be developed and maintained particularly between pharmacist and patients. One study identified that accessibility, respect, communication skills and a friendly behavior from pharmacists are considered as trust enhancing factors [36]. The average time of consultation was from 1 to 5 min, however, the lack of time due to the economic situation and the decrease in the number of employees in the pharmacies are examples of obstacles in front of good counseling to patients and was reported in one previous study [6]; this was also similar to a study conducted in Portuguese community pharmacies with a mean duration of 3.98 min per interaction between the patient and community pharmacist [37].The inadequate perception of customers meets also the results of a regional study in Baghdad that recommended allocation of more time for patient counseling, helping patients to manage their medications and extend their working hours to meet customer needs [38].

In regard to storage requirements, participants expressed a very positive opinion. Results were similar to the pilot study conducted by Badro et al. 2020 showing that medicines were protected from direct exposure to sunlight, presence of a functional cooling and heating system, and absence of any signs of leaks in the roof which in turn can help preserve the quality of pharmaceutical products and ensures patient safety. Younger patients and those with high income had a positive perception towards pharmacy services; these findings do not correlate with the results of a study conducted in Lebanon by Iskandar et al. 2017. This may be possible due to the current financial crisis facing the Lebanese population. In addition, the unemployed participants showed a negative perception.

An interesting information in this study is that most participants confessed that they do not know if there is a licensed pharmacist as long as the pharmacy is open (73.1%); this can be considered as a trust diminishing factor and defined as lack of adopting rules and regulatory requirements for the profession [28]. Finally, and despite all the above issues, pharmacy services were enhanced during the COVID-19 crisis because 67.1% of respondents preferred consulting most of the time the community pharmacist instead of visiting primary health care centers, doctor’s private clinic and hospitals. These results revealed that community pharmacists are on the frontline and had a major role during the crisis [39].

### Limitations

This study had several limitations. The main limitation was the current socio-economic crisis that may influence the public opinion regarding the GPP performed. Some of the questions may be led by memory biases which might under or overestimate public opinion. Also, some of the respondents might be related to health care sectors, which may also influence the results positively. A selection bias is possible, and due to the convenience sampling technique used; the results cannot be generalizable. The original survey tool was created by the OPL, but it has not been validated on a second sample. The Cronbach’s alpha values were less than 0.7; this might be because the general population did not really understand the questions asked since they were originally tailored to pharmacists.

## Conclusion

The level of GPP perceived by the public was inadequate. Perception towards GPP differs in the absence and presence of community pharmacists in terms of quality of services. The public reported huge shortage of medicines and closure of pharmacies. Therefore, community pharmacists are encouraged to maintain high standards and abide by the basic requirements to gain more public trust and confidence and aid in handling better quality of care. Such requirements can include spending more time on counseling, helping patients to manage their medications depending on their medical status and history; health promotion and therapeutic education efforts are also recommended especially when patients ask to buy antibiotics without a medical prescription. The MOPH is highly urged to help in ensuring the medicinal products either by importation or by putting a plan to manufacture locally and to enforce the laws by the presence of a licensed pharmacist as long as the pharmacy is open. It is also suggested that the MOPH and the OPL conduct awareness campaigns to the public about the role of the pharmacist. Finally, regular assessment should be carried to identify customer perspectives and satisfaction towards community pharmacy services.

## Data Availability

All data generated or analyzed during this study are included in this published article.
